# A Virtual National Diversity Mentoring Initiative to Promote Inclusion in Emergency Medicine

**DOI:** 10.5811/westjem.59666

**Published:** 2023-07-12

**Authors:** Tatiana Carrillo, Lorena Martinez Rodriguez, Adaira Landry, Al’ai Alvarez, Alyssa Ceniza, Riane Gay, Andrea Green, Jessica Faiz

**Affiliations:** * University of Pennsylvania, Department of Emergency Medicine, Philadelphia, Pennsylvania; † University of South Florida, Department of Emergency Medicine, Tampa, Florida; ‡ Harvard Medical School, Department of Emergency Medicine, Boston, Massachusetts; § Stanford University, Department of Emergency Medicine, Stanford, California; ¶ Texas Tech University Health Sciences Center/University Medical Center of El Paso, El Paso, Texas; || American College of Emergency Physicians, Dallas, Texas; # National Clinician Scholars Program, VA Greater Los Angeles Healthcare System and UCLA, Los Angeles, California

## Abstract

**Introduction:**

Trainees underrepresented in medicine (URiM) face additional challenges seeking community in predominantly white academic spaces, as they juggle the effects of institutional, interpersonal, and internalized racism while undergoing medical training. To offer support and a space to share these unique experiences, mentorship for URiM trainees is essential. However, URiM trainees have limited access to mentorship from URiM faculty. To address this gap, we developed a national virtual mentoring program that paired URiM trainees interested in emergency medicine (EM) with experienced mentors.

**Methods:**

We describe the implementation of a virtual Diversity Mentoring Initiative (DMI) geared toward supporting URiM trainees interested in EM. The program development involved 1) partnering of national EM organizations to obtain funding; (2) identifying a comprehensive platform to facilitate participant communication, artificial intelligence-enabled matching, and ongoing data collection; 3) focusing on targeted recruitment of URiM trainees; and (4) fostering regular leadership meeting cadence to customize the platform and optimize the mentorship experience.

**Conclusion:**

We found that by using a virtual platform, the DMI enhanced the efficiency of mentor-mentee pairing, tailored matches based on participants’ interests and the bandwidth of mentors, and successfully established cross-institutional connections to support the mentorship needs of URiM trainees.

## BACKGROUND

Trainees underrepresented in medicine (URiM)* face challenges seeking community in predominantly White academic spaces, as they juggle the effects of institutional, interpersonal, and internalized racism throughout medical training.^1^–^12^ These effects can contribute to increased experiences of the imposter phenomenon among URiM trainees, which can negatively affect performance.^13^–^20^ Lack of social networks within medicine and informal knowledge due to differences in parental education and resources contribute to barriers to inclusion and career success.^21^–^25^ To offer support and a space to share these unique experiences, mentorship for URiM trainees is essential in academic medicine, in addition to its benefits of promoting greater career satisfaction, productivity, and physician wellness.^26^–^36^

However, there is a scarcity of mentorship for URiM trainees, who especially benefit from faculty of diverse gender identities and racial and ethnic backgrounds.^37^,^38^ URiM faculty are instrumental in promoting inclusive training environments and have been shown to increase residency programs’ ability to recruit Black, Latinx, and Native American residents and increase graduation rates among URiM students.^39^–^43^ Unfortunately, this mentorship is limited by the small number of URiM faculty, who already take on a disproportionate burden of mentorship.^44^–^47^ This minority tax contributes to increased burnout, decreased productivity, and decreased career promotion.^48^–^51^

To address this gap, we developed a national virtual mentoring program that strategically pairs URiM trainees interested in EM with mentors familiar with medical education and career development. We describe how to implement a model to expand mentorship access for URiM trainees and advance representation, inclusion, and belonging in EM.

## OBJECTIVES

The virtual Diversity Mentoring Initiative (DMI) aimed to (1) provide an accessible and safe environment for URiM participants to share experiences; (2) connect with other URiM trainees and mentors in EM; and (3) provide practical strategies to thrive personally and professionally as future emergency physicians. We measured these objectives by self-reported surveys regarding satisfaction with the DMI and mentees’ ability to achieve mentorship goals. This study was deemed exempt from institutional review board (IRB) review by the Stanford IRB.

## INNOVATION DESIGN

The American College of Emergency Physicians (ACEP) and the Emergency Medicine Residents’ Association (EMRA) partnered to form the DMI in September 2019. Faculty and trainees beta-tested several virtual mentoring platforms. The primary need for a virtual format was the ability to scale the DMI for large cohorts by enhancing the efficiency of matching mentor-mentee pairs, as opposed to manual pairing. Other important features included a user-friendly interface, a single-sign-on feature using existing ACEP logins, and the ability to collect data on participant interactions and mentor-mentee pairs for reportable outcomes.

We chose the Chronus software (Chronus LLC, Bellevue, WA) for its comprehensive platform with the ability to perform artificial intelligence-enabled matching of mentor-mentee pairs, enroll participants, facilitate participant communication, and collect longitudinal data.^52^ Chronus included an account manager that generated reports, oriented users, and troubleshooted issues, beyond the information technology support that most other platforms offered.

Upon program initiation, mentors and mentees completed a questionnaire in the Chronus platform indicating their goals, areas of expertise and, for mentors their number of desired mentees. Chronus then suggested pairings based on responses. Mentees had the final choice to select their mentor from a short list.

Three mentorship cycles were completed: August 2020–February 2021, March 2021–September 2021, and October 2021–June 2022. We advertised through Twitter, EM medical education networks (e.g., Council of Residency Directors in EM), the EMRA Diversity and Inclusion committee, and targeted outreach to Historically Black Colleges and Universities’ EM interest groups to recruit participants.

To supplement individual mentor-mentee relationships, nine 60-minute educational and networking events for program participants, called “#MixMatchMingle,” were held throughout the three cycles to create a communal space to share experiences. The format of the #MixMatchMingle events ranged from invited guest speakers to facilitator-led discussions on a topic. Event themes are listed in Table 1 and were chosen by the leadership team, who had various levels of training and faculty experience, diverse racial and ethnic backgrounds, members who were first-generation in medicine, and faculty specializing in medical education. By strategically pairing mentors and URiM mentees based on their goals and interests and fostering a wider community with the #MixMatchMingle curriculum, DMI uses mentorship as a pedagogical approach to EM career guidance. It also leverages strategies grounded in social capital and social network theory to address potential disparities in informal networks and knowledge in academia.^53^,^54^

Monthly quantitative and qualitative data were collected via Chronus throughout each cycle to measure program efficacy and inform subsequent iterations (full survey in Supplemental Material). Incremental iterations of workflow and customization of the platform were necessary as unexpected challenges arose. For example, when the number of enrollees did not match the number of participants paired, we found that it was largely due to lack of participant profile completion. Thus, we adjusted automated e-mail reminders and more explicitly communicated the importance of profile completion to participants.

## IMPACT/EFFECTIVENESS

A total of 87 mentors and 270 mentees participated in the DMI. The average mentor to mentee ratio was 1 to 1.6. Anonymous surveys were distributed to participants. Of the 270 mentees, 215 (79.6%) responded to the end-of-cohort survey. Successful implementation of the DMI required funding and staff support, platform troubleshooting, and early scheduling of events for participants. We leveraged an existing partnership between ACEP and an acute care staffing company Vituity (CEP America, Inc, Emeryville, CA) to fund Chronus. Administrative and physician program leaders met biweekly to discuss data generated by the platform, troubleshoot issues with mentor-mentee pairing, and design the #MixMatchMingle curriculum.

The virtual mentoring format successfully provided access to mentors dedicated to increasing representation in medicine. Travel limitations during the pandemic especially impacted medical students enrolled at institutions without associated EM programs.^55^–^57^ One participant noted, *“I really appreciated the virtual format, especially since my mentor is across the country. It made for a unique opportunity that wouldn’t have been possible otherwise.”* The virtual format lent itself to participant flexibility, with another mentee noting, *“The online format made it convenient for me to participate despite my hectic and unsure schedule.”*

In the survey distributed upon cohort completion, 105 of 117 (89.7%) mentee respondents rated that they were “satisfied” or “strongly satisfied” with their mentorship, and 107 of 117 (91.5%) mentee respondents stated that they achieved the goals set for their mentorship relationship. Mentees described the impact of their mentoring relationships on both personal and professional levels, with one sharing, *“[Mentor name] really changed the game for me. I was not sure if I wanted to continue with medical school. Now I know that I not only want to become a doctor, but I want to be in emergency medicine for sure.”* Another participant noted, *“[Mentor name] is amazing and not only do I feel like I have a mentor for life, but I’ve also gained a good friend.”*

The fluidity of the mentorship structure had mixed feedback. While some participants enjoyed organically building relationships with their mentors through more casual interactions, others suggested benefiting from a more structured curriculum and concrete goal setting. We found that engagement with the #MixMatchMingle events waned as the pandemic continued, which may in part have been due to “Zoom fatigue.”^58^,^59^ Given that participants cited positive experiences during one-on-one mentor-mentee meetings, we reallocated efforts by decreasing the number of program-wide events.

We were encouraged that the proportion of URiM mentors was disproportionately higher than their representation in EM. Balancing the benefits of having racially concordant mentors with mitigating the minority tax on these mentors was challenging.^60^,^61^ However, compensation and academic incentives for mentor participation could be effective.^62^–^64^

## LIMITATIONS

This study had limitations. First, physician leaders did not have protected time for this pilot initiative, limiting their availability for meetings and recruitment efforts. Dedicated administrative staff and protected time for physician leaders is critical for program sustainability. Furthermore, a mentorship platform with automated matching may be cost-prohibitive for many institutions, at a cost of $42,000 annually.

Because participants were only able to select one race or ethnicity, we were unable to capture the perspectives of multiracial participants. Finally, the data did not include intersectional identities such as sexual orientation, religion, disability status, and being first-generation, which may have contributed to participants’ experiences.^65^ With added support, targeted recruitment could have been expanded to Hispanic-serving institutions and other national student organizations.

## CONCLUSION

The national virtual Diversity Mentoring Initiative successfully provided access to cross-institutional connections and supported the specific mentorship needs of URiM trainees. Using a virtual platform enhanced the efficiency of mentor-mentee pairing and allowed for tailored matches based on participants’ interest and bandwidth of mentors. The majority of mentees reported satisfaction with their mentor relationships, highlighting support for their personal and professional growth. Support from national organizations to fund virtual DMI for EM interest groups and residency programs can bridge geographical gaps in mentorship and sponsorship. We hope that this model is translated beyond EM and inspires the launch of personalized mentorship resources for URiM trainees to further inclusion and a sense of belonging, while broadly advancing representation in medicine.

## Supplementary Information



## Figures and Tables

**Table 1:** Topics of #MixMatchMingle events.

Cohort Introduction (orientation to Chronus, The Why’s and How’s of Effective Mentorship)
Addressing Impostor Phenomenon
Residency Application Preparation and Tips
COVID-19 Pandemic Reflections and Practicing Self-Compassion
Thriving Professionally and Financially: Building your Wealth in the Stock Market
“Life Hacks” and Time Management
Transitions in Medicine and the Role of Mentorship
Mentor/Mentee-ship at Different Career Levels
Pathways to Leadership in EM

*COVID-19*, coronavirus disease 2019; *EM*, emergency medicine.

**Table 2:** Characteristics of unique participants of the virtual Diversity Mentoring Initiative across three cohorts (August 2020–June 2022).

	Total N (%)	Mentors	Mentees
N (%)	357	87 (24.4)	270 (75.6)
Gender			
Men	96 (26.9)	41 (47.1)	55 (20.4)
Women	251 (70.3)	42 (48.3)	209 (77.4)
Non-Binary	6 (1.7)	1 (1.2)	5 (1.9)
Transgender	3 (0.8)	2 (2.3)	1 (0.4)
Undisclosed	1 (0.3)	1 (1.2)	0
Level of Training			
MS Year 1 or 2	92 (25.8)	0	92 (34.1)
MS Year 3 or 4	135 (37.8)	0	135 (50.0)
PGY1	36 (10.1)	15 (17.2)	21 (7.8)
PGY2	10 (2.8)	3 (3.4)	7 (2.6)
PGY3	15 (4.2)	7 (8.0)	8 (3.0)
PGY4	6 (1.7)	5 (5.7)	1 (0.4)
Fellow	19 (5.3)	18 (20.7)	1 (0.4)
Attending (1–5 years post-residency)	14 (3.9)	13 (14.9)	1 (0.4)
Attending (5+ years post-residency)	6 (1.7)	6 (6.9)	0
Other*	24 (6.7)	20 (23.0)	4 (1.5)
Race or Ethnicity			
Asian	63 (17.6)	11 (12.6)	52 (19.3)
Black	121 (33.9)	24 (27.6)	97 (35.9)
Latino/a	74 (20.7)	13 (14.9)	61 (22.6)
Middle Eastern	14 (3.9)	4 (4.6)	10 (3.7)
Native American	5 (1.4)	0	5 (1.9)
Native Hawaiian or Pacific Islander	2 (0.6)	1 (1.2)	1 (0.4)
White	60 (16.8)	33 (37.9)	27 (10)
Other	18 (5.0)	1 (1.2)	17 (6.3)

* Participants who listed “other” for level of training cited roles such as MD-PhD, MBBS, osteopathic medical student, and chief resident.

*MS*, medical student; *PGY*, postgraduate year.

## Footnotes

* The Association of American Medical Colleges (AAMC) defined the term underrepresented minority (URM) to reflect the racial groups of Black, Mexican-American, mainland Puerto Rican, and Native American (American Indian and natives of Alaska and Hawaii). In 2003, to encompass the racial and ethnic populations within medicine who are underrepresented when compared to their respective numbers in the context of the greater population, this was further clarified to “underrepresented in medicine” (URiM or UIM). Although we recognize the use of URiM or UIM interchangeably, for consistency, we have used the term URiM throughout this paper.
