# Supplementary material for: A Virtual National Diversity Mentoring Initiative to Promote Inclusion in Emergency Medicine
Source: West J Emerg Med. 2023 Jul 12;24(4):662–7. doi: 10.5811/westjem.59666 (PMC10393448; doi:10.5811/westjem.59666)
Supplement: Supplementary file 1 [file wjem-24-662-s001.docx]

**Supplement 1.** 30-Day Checkpoint Participant Survey

| 30 Day Checkpoint Survey Questions |
| --- |
| How easy is the software platform to use?  *Scale: 5 (Very easy) - 1 (Very difficult)* |
| How comfortable do you feel with your mentoring partner?  *Scale: 5 (Very comfortable) - 1 (Not comfortable)* |
| Would you like to be rematched? |
| Have you started setting goals for your mentoring connection? |
| Please share any other specific feedback you have about your experience so far. |

**Supplement 2.** 90-Day Checkpoint Participant Survey

| 90 Day Checkpoint Survey Questions - Mentee |
| --- |
| Please rate your progress towards completing your goals.  *Scale: 5 (Strongly satisfied), 4 (Satisfied), 3 (Neutral), 2 (Dissatisfied), 1 (Strongly dissatisfied)* |
| Please rate your overall satisfaction so far with your mentoring partnership  *Scale: 5 (Strongly satisfied), 4 (Satisfied), 3 (Neutral), 2 (Dissatisfied), 1 (Strongly dissatisfied)* |
| Please explain your above answer. |
| What is going well so far? |
| What could be improved? |

**Supplement 3.** 180-Day Checkpoint Participant Survey

| 180 - Day Closure Survey - Mentee |
| --- |
| How would you rate your overall satisfaction with your mentorship?  *Scale: 5 (Strongly satisfied), 4 (Satisfied), 3 (Neutral), 2 (Dissatisfied), 1 (Strongly dissatisfied)* |
| What is the primary reason for your above rating? |
| Did you achieve the goals you set for this mentorship?  *Scale: 5 (Agree), 4 (Agree), 3 (Neutral), 2 (Disagree), 1 (Disagree)* |
| What is the reason(s) for your above answer? |
| How satisfied were you with your mentoring partner?  *Scale: 5 (Strongly satisfied), 4 (Satisfied), 3 (Neutral), 2 (Dissatisfied), 1 (Strongly dissatisfied)* |
| What is the reason(s) for your above answer? |
| How many times did you meet with your mentoring partner? |
| Based on the competencies you identified for improvement in your profile, which do to feel you have improved upon because of this mentorship? |
| Based on the areas you identified that mentoring could help you, which do to feel have improved upon because of this mentorship? |
| Based on the skills you identified for improvement in your profile, which do to feel you have improved upon because of this mentorship? |
